# A bronze statue of the acromegalic giant Rigardus Rijnhout

**DOI:** 10.1007/s40618-023-02139-8

**Published:** 2023-06-17

**Authors:** W. W. de Herder

**Affiliations:** Sector of Endocrinology, Department of Internal Medicine, Rg520, Dr. Molewaterplein 40, 3015 GD Rotterdam, The Netherlands

**Fig. 1:**
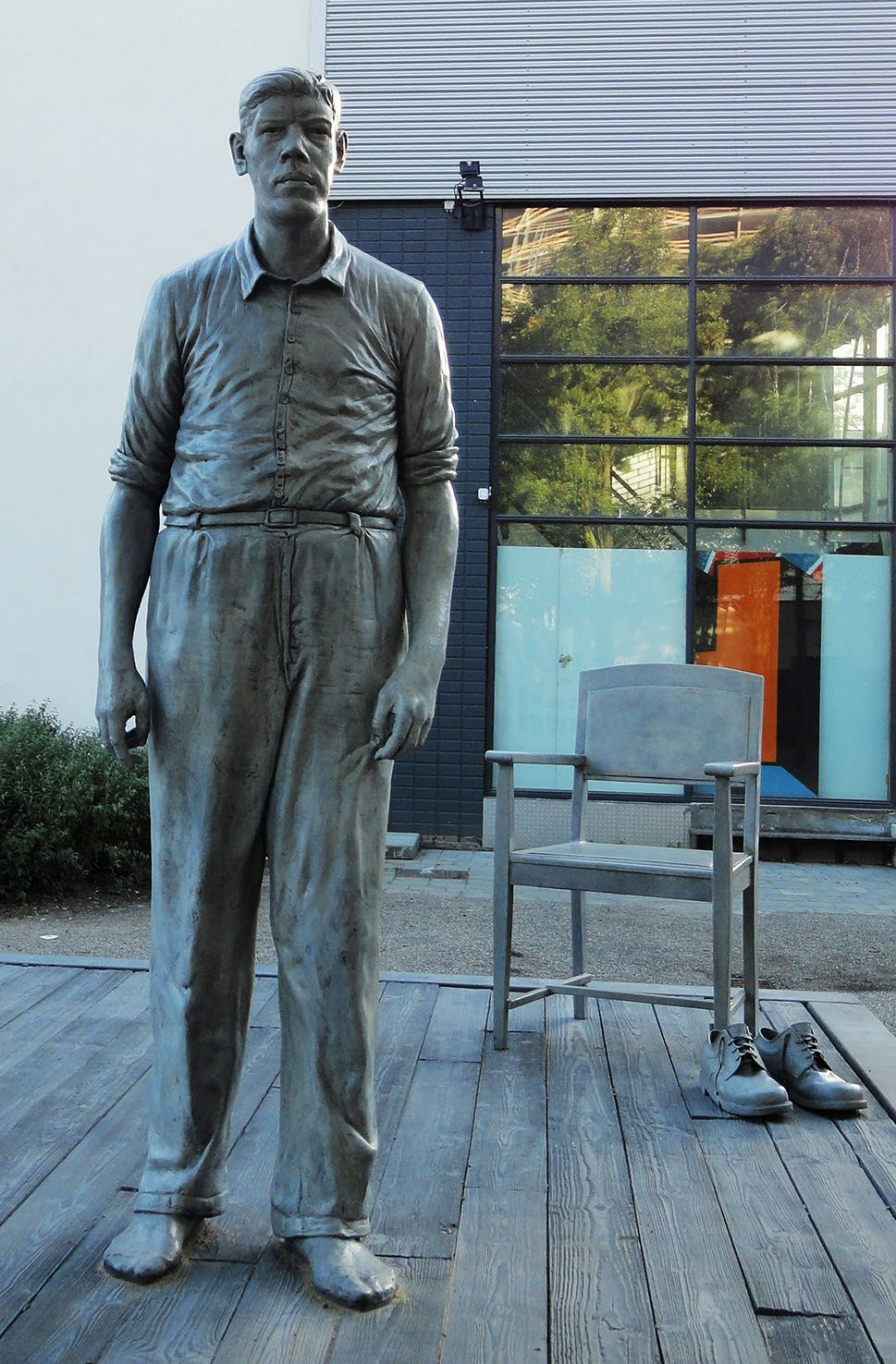
Photograph of the life-size bronze statue and bronze shoe replicas of Rigardus Rijnhout (1922–1959) in Rotterdam, the Netherlands. Photograph taken by Wouter de Herder

**Fig. 2:**
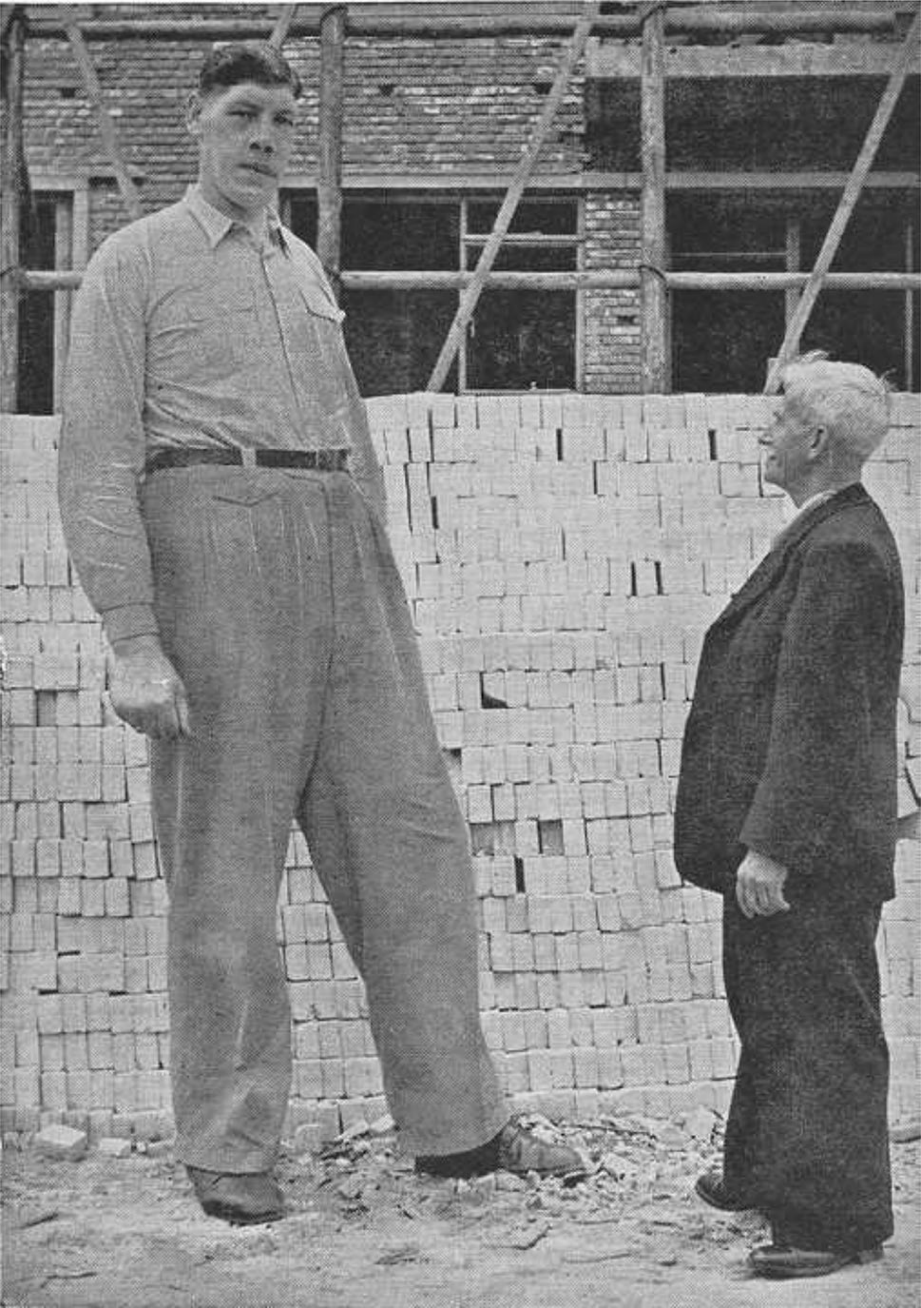
Photograph of Rigardus Rijnhout (1922–1959) at the age of 31 years in Rotterdam, the Netherlands next to a person of ordinary size. Picture postcard from the collection of Wouter de Herder

Bronze life-size statue by the Dutch sculpturer Herman Lamers (1954, https://www.hermanlamers.nl/) of Rigardus Rijnhout (“de Reus van Rotterdam”, “the Rotterdam giant”), flanked by a pair of his replicated bronze shoes and a chair, was erected in Rotterdam, the Netherlands in 2011 (Figs. 1, 2).

The acromegalic giant, Rigardus Rijnhout was born on the 21th of April 1922 in Rotterdam, the Netherlands. His final height was 2.375 m. And the length of his feet was 36.0 cm. He developed normally until the age of 3. At the age of 13, his height was already 1.90 m. At the age of 21, he was treated with external pituitary radiotherapy. He stopped growing at the age of 24. He developed hypopituitarism, for which he received desiccated thyroid extract and testosterone proprionate injections. He died at the age of 36, on the 13th of April 1959, as a result of a (uro-)sepsis after being hospitalized for 141 days under the care of the endocrinologist Andries Querido (1912–2001) in the Leiden University Hospital, Leiden, the Netherlands [1, 2].

At least three other bronze (life-size) statues of once famous acromegalic giants exist. In this journal there was already a report on the bronze statue of Robert Pershing Wadlow (2.72 m., 1918–1940) in Alton, Illinois, USA, which was created in 1985 by the American sculpturer Edward Engelhardt Giberson (1948) [3]. A bronze life-size statue of Fermin Arrudi Urieta (223.5 cm, 1870–1913, “el Gigante de Sallent”, “the Sallent giant”, “the Aragonese giant”) [4, 5] was created in 2014 by the Spanish sculpturer Jaqués Pedro J Larraz and can be found in Sallent de Gállego, Spain. A bronze life-size statue of Jose Calderon Torres (235 cm, 1915–1973, “the Tampico Giant”) [6], seated on a bronze bench was created in 1975 by the Mexican sculpturer Víctor Hugo Yáñez Piña (1967) and named “Peptito el Terrestre”. It can be found in Tampico, Colonia Arenal, Mexico.
